# Progress in Shortening Treatment Courses for Bone Metastases in a Statewide Quality Consortium

**DOI:** 10.1016/j.adro.2026.102008

**Published:** 2026-02-13

**Authors:** Luke M. Higgins, Huiying (Maggie) Yin, Kent Griffith, Jumoke Johnson-Olokesusi, Kelly C. Paradis, Amit K. Bhatt, Lana Critchfield, Kaitlyn Baldwin, Vrinda Narayana, Howayda Messiha, Jennifer Davis, Mohamad Fakhreddine, Eyad Abu-Isa, James A. Hayman, Michigan Radiation Oncology Quality Consortium as part of the Blue Cross Blue Shield of Michigan and Blue Care Network of Michigan Value Partnerships Program

**Affiliations:** a Department of Radiation Oncology, University of Michigan, Ann Arbor, Michigan; b Department of Biostatistics, University of Michigan, Ann Arbor, Michigan; c Michigan Radiation Oncology Quality Consortium Coordinating Center, Ann Arbor, Michigan; d Karmanos Cancer Institute at McLaren Greater Lansing, Lansing, Michigan; e Department of Radiation Oncology, University of Michigan Health – West, Grand Rapids, Michigan; f Department of Radiation Oncology, Munson Medical Center, Traverse City, Michigan; g Henry Ford Health Providence Hospital, Novi, Michigan; h West Michigan Cancer Center, Kalamazoo Michigan

## Abstract

**Purpose:**

A large body of research has studied various fractionation regimens for radiation therapy (RT) targeting bone metastases, with evidence that courses of 5 or fewer fractions are isoeffective compared with longer courses. We analyzed practice patterns within a statewide quality consortium following the implementation of quality measures promoting single-fraction RT (SFRT) for uncomplicated metastases and ≤5 fractions for all metastases.

**Methods and Materials:**

Consecutive patients receiving RT for bone metastasis from primary breast, lung, melanoma, prostate, or renal cancer(s) between March 1, 2018, and December 31, 2024, were prospectively enrolled in a statewide quality consortium. SFRT and ≤5-fraction quality metrics were implemented on January 1, 2020, and January 1, 2022, respectively. Patient, treatment, physician, and facility characteristics were collected, and multivariable logistic regression, with and without random center intercepts, was used to account for clustering by center and to assess associations with metric adherence.

**Results:**

In total, 4477 patients were enrolled and received 6733 RT treatment plans, with 1105 patients receiving 1465 plans for uncomplicated metastases and 3247 patients receiving 4832 nonstereotactic body RT plans. The use of SFRT and ≤5-fraction RT for uncomplicated and any metastases increased from 17.8% to 38.8% and from 44.2% to 63.9%, respectively, after the implementation of quality measures. In both models, later years of treatment, longer distance to the treating facility, higher baseline fatigue, treatment site other than the spine, and fewer physician years in practice predicted shorter treatment courses. Patients with >1 site treated for uncomplicated metastases had lower odds of receiving SFRT. Forward planning, uncomplicated metastasis, retreatment, palliative intent, age ≥80 years, and an Eastern Cooperative Oncology Group performance status ≥2 independently predicted receipt of ≤5 fractions.

**Conclusions:**

Our efforts to shorten treatment courses for bone metastases have been successful. The number and variety of factors that predict the use of shorter courses reflect the complexity of clinical decision-making in the treatment of bone metastases.

## Introduction

National and international guidelines support the use of various fractionation regimens for radiation therapy (RT) of bone metastases.^1^^,^^2^ The ESTRO-ACROP (European Society for Radiotherapy and Oncology Advisory Committee on Radiation Oncology) guidelines recommend single-fraction RT (SFRT) for uncomplicated bone metastases.^3^ RT is commonly used for palliation of painful bone metastases, and a variety of effective fractionation approaches have been reported in the literature, with some studies reporting a higher retreatment rate with SFRT.^4^, ^5^, ^6^ Growing evidence supports the utility of RT for select asymptomatic bone metastases to prevent skeletal events or for patients with oligometastatic disease, often with a stereotactic ablative approach.^7^, ^8^, ^9^, ^10^ The selection of fractionation for RT of bone metastases is complex, considering multiple patient-specific factors, including treatment indication, anatomic location, and prognosis, among others. Treatment with fewer fractions can conserve health system(s) and patient resources and may reduce the environmental impact of RT.^11^, ^12^, ^13^, ^14^

The Michigan Radiation Oncology Quality Consortium (MROQC) is a collaborative effort among academic and community radiation oncology centers in Michigan that prospectively enrolls patients receiving RT for select indications and collects relevant data.^15^^,^^16^ Within MROQC, we previously reported real-world data demonstrating heterogeneity in the use of fractionation regimens, including persistent but infrequent use of extended fractionation RT (EFRT; >10 fractions) and the use of SFRT in a minority of cases.^17^^,^^18^ In previously reported efforts, after developing and implementing quality metrics to promote high-value radiation oncology approaches and reduce the use of EFRT, we observed a lower rate of EFRT; however, only 12% of plans delivered 8 Gy SFRT, and nearly half of the plans delivered 10 fractions of RT in this series.^19^ National data highlight similar trends in the reduction of EFRT, but a limited increase in the use of SFRT.^20^

MROQC quality metrics were subsequently introduced to promote the use of SFRT for uncomplicated bone metastases and of 5 or fewer fractions for any bone metastasis. We hypothesized that practice patterns would remain varied, given the complexity of fractionation selection, with the proportion of patients receiving SFRT and fewer than 5 fractions anticipated to increase with the introduction of respective quality metrics.

## Methods and Materials

Consecutive patients receiving RT for bone metastases from primary breast, lung, melanoma, prostate, or renal cancer(s) between March 1, 2018, and December 31, 2024, were prospectively enrolled in the statewide MROQC database from participating centers.^15^ Data collection instruments measuring patient, treatment, physician, and facility characteristics were collected prospectively via surveys distributed to both patients and clinicians. Treatment characteristics, including treatment sites, treatment intent, baseline symptoms, and the presence of spinal cord or cauda equina compression, were physician-assessed. Collected responses were manually entered into an electronic database. Consecutive patients enrolled in the database were included, regardless of insurance status or insurer. The work of MROQC is designated as quality improvement and has been reviewed by the University of Michigan institutional review board, which has determined that it is exempt from further oversight.

Quality metrics were introduced to participating facilities as a component of an incentive structure through Blue Cross Blue Shield of Michigan (BCBSM), including a facility-level pay-for-performance program, a facility-level prior authorization “gold card” program (facilities with “gold card” status are exempt from BCBSM’s radiation oncology prior authorization program), and a physician-level value-based reimbursement program. Multiple quality initiatives were used in parallel across selected disease sites. Inclusion metrics and exact metric weights varied by year, and quality metrics across multiple disease sites were included in incentive programs.

A quality metric promoting the use of SFRT for uncomplicated bone metastases was initiated on January 1, 2020. Uncomplicated metastases were defined as painful, not previously irradiated, and not associated with spinal cord or nerve root compression, fracture, surgery, or a soft tissue component. A provider-facing knowledge transfer project, including an educational video followed by a facilitated discussion, was conducted at 25 participating centers between 2022 and 2023 to promote SFRT.^21^ A quality metric promoting the use of ≤5-fraction RT for all bone metastases was initiated on January 1, 2022, without penalty for stereotactic body RT (SBRT) plans, in an incentive structure. An example annual quality measure benchmark aims to achieve ≥75% of bone metastases treated in ≤5 fractions at the facility level and ≥45% of patients with uncomplicated metastases treated with SFRT across the consortium.

Patient, treatment, physician, and facility characteristics were summarized using descriptive statistics, including adherence to quality metrics and changes in the use of SFRT and ≤5-fraction RT over time. Characteristics used were assessable at the time of receipt of RT. Oncologic outcomes were not assessed.

The use of SFRT and ≤5-fraction RT was assessed for association with the collected variables among patients with uncomplicated metastases and among all enrolled patients, respectively. For analysis of ≤5-fraction RT, SBRT plans were excluded from analysis, given that all were ≤5 fractions. If multiple areas were treated in a treatment course, the dose and fractionation with the largest number of fractions were used for description and analysis. Planning modality was collected, including forward planning (manually selected beam direction, shape, and weight), inverse planning (plans optimized from treatment goals for target dose and organ-at-risk [OAR] avoidance using commercially available software), or hybrid planning (a combination of the 2 strategies).

Multivariable logistic regression with and without random center intercepts to account for clustering was generated, with significance defined as *P* < .05. Year of treatment was assessed both as a continuous variable and as defined categorical time periods before and after the launch of applicable quality initiatives for inclusion in the models. The distance a patient traveled to the treating facility was estimated using the centroids of the U.S. ZIP code(s) of the patient’s residence and the treating facility. Distance from the treating facility was reported by terciles in the overall cohort, but a continuous variable with a unit of 10 miles was used for multivariable analyses. Physicians’ years of practice were reported categorically in 10-year intervals. Treatment intent (given that all uncomplicated metastases were treated for palliation) and retreatment (defined as treatment to the same site by the treating physician) were excluded from models for uncomplicated metastases, given that they violated our definition of uncomplicated metastases.

Statistical analysis was completed using SAS version 9.4 (SAS Institute).

## Results

During the observation period, 4477 patients were enrolled and received 6733 RT plans. Uncomplicated metastases were observed in 1105 patients receiving 1465 plans and were included in the SFRT analysis. After excluding SBRT plans, 3247 patients receiving 4832 plans were included in the analysis of plans delivering ≤5-fraction RT for any bone metastasis. SBRT use increased over time, from 8.6% of cases in the consortium in 2018 to 29.8% in 2024. Patient, treatment, physician, and facility characteristics of both cohorts are described in Table 1, with many of the characteristics expectedly imbalanced between patients receiving SFRT and those receiving ≤5-fraction RT. All 29 participating centers enrolled patients during the observation period, and the number of enrolling providers per center ranged from 1 to 23. The number of participating centers varied from year to year.

**Table 1:** Clinical, patient, and facility characteristics for all patients enrolled with exclusion of stereotactic body radiation therapy plans

	All plans	Plans > 5 fractions	Plans ≤ 5 fractions	*P* value	Uncomplicated metastases	Plans > 1 fraction	Plans = 1 fraction	*P* value
Variable	N (column %)	N (row %)	N (row %)		N (column %)	N (row %)	N (row %)	
N plans	4832	2215	2617		1465	968	497	-
N patients	3247	1775	1777		1105	761	401	-
**Patient/clinical characteristics**								
Age				<.0001				.35176
≤59	1135 (23.5)	560 (49.3)	575 (50.7)	-	339 (23.1)	235 (69.3)	104 (30.7)	-
60-69	1597 (33.1)	752 (47.1)	845 (52.9)	-	454 (31.0)	303 (66.7)	151 (33.3)	-
70-79	1371 (28.4)	634 (46.2)	737 (53.8)	-	428 (29.2)	277 (64.7)	151 (35.3)	-
80+	729 (15.1)	269 (36.9)	460 (63.1)	-	244 (16.7)	153 (62.7)	91 (37.3)	-
Sex				.2171				.00172
Female	2401 (49.7)	1122 (46.7)	1279 (53.3)	-	744 (50.8)	520 (69.9)	224 (30.1)	-
Male	2431 (50.3)	1093 (45.0)	1338 (55.0)	-	721 (49.2)	448 (62.1)	273 (37.9)	-
Race				.418				.34464
White	3998 (82.7)	1824 (45.6)	2174 (54.4)	-	1210 (82.6)	795 (65.7)	415 (34.3)	-
Black	609 (12.6)	284 (46.6)	325 (53.4)	-	187 (12.8)	130 (69.5)	57 (30.5)	-
Other	155 (3.2)	79 (51.0)	76 (49.0)	-	36 (2.5)	20 (55.6)	16 (44.4)	-
Unknown	70 (1.4)	28 (40.0)	42 (60.0)	-	32 (2.2)	23 (71.9)	9 (28.1)	-
Primary cancer site				.003				.00228
Breast cancer	1489 (30.8)	725 (48.7)	764 (51.3)	-	485 (33.1)	352 (72.6)	133 (27.4)	-
Melanoma	80 (1.7)	30 (37.5)	50 (62.5)	-	10 (0.7)	8 (80.0)	2 (20.0)	-
NSCLC	1445 (29.9)	646 (44.7)	799 (55.3)	-	418 (28.5)	275 (65.8)	143 (34.2)	-
Prostate cancer	1244 (25.7)	566 (45.5)	678 (54.5)	-	403 (27.5)	246 (61.0)	157 (39.0)	-
Renal cell cancer	352 (7.3)	169 (48.0)	183 (52.0)	-	67 (4.6)	40 (59.7)	27 (40.3)	-
SCLC	222 (4.6)	79 (35.6)	143 (64.4)	-	82 (5.6)	47 (57.3)	35 (42.7)	-
Distance from site (miles)				.2478				.22034
First tercile (0-7)	1685 (35.0)	799 (47.4)	886 (52.6)	-	535 (36.6)	362 (67.7)	173 (32.3)	-
Second tercile (8-19)	1616 (33.6)	732 (45.3)	884 (54.7)	-	508 (34.8)	343 (67.5)	165 (32.5)	-
Third tercile (>19)	1515 (31.5)	676 (44.6)	839 (55.4)	-	417 (28.6)	262 (62.8)	155 (37.2)	-
Planning type				<.0001				.00331
Forward planning	4340 (90.0)	1930 (44.5)	2410 (55.5)	-	1271 (86.9)	821 (64.6)	450 (35.4)	-
Hybrid technique	2 (0.0)	1 (50.0)	1 (50.0)	-	-	-	-	-
Inverse planning	478 (9.9)	276 (57.7)	202 (42.3)	-	191 (13.1)	144 (75.4)	47 (24.6)	-
Uncomplicated bone metastasis				<.0001				
Complicated	3346 (73.2)	1682 (50.3)	1664 (49.7)	-	-	-	-	-
Uncomplicated	1222 (26.8)	406 (33.2)	816 (66.8)	-	1465 (100)	968 (66.1)	497 (33.9)	-
Opioids use				.0217				.43648
No	1183 (27.3)	588 (49.7)	595 (50.3)	-	364 (27.1)	249 (68.4)	115 (31.6)	-
Yes	3153 (72.7)	1444 (45.8)	1709 (54.2)	-	981 (72.9)	649 (66.2)	332 (33.8)	-
Primary treatment intent				<.0001				-
Palliation of existing symptoms	3267 (68.2)	1299 (39.8)	1968 (60.2)	-	1465 (100.0)	968 (66.1)	497 (33.9)	-
Curative intent or improvements in PFS	69 (1.4)	32 (46.4)	37 (53.6)	-	-	-	-	-
Durable local control	859 (17.9)	502 (58.4)	357 (41.6)	-	-	-	-	-
Treatment of existing pathological fracture	314 (6.6)	198 (63.1)	116 (36.9)	-	-	-	-	-
Treatment of spinal cord compression	279 (5.8)	166 (59.5)	113 (40.5)	-	-	-	-	-
Anatomic site treated				<.0001				<.0001
Hip/pelvis/femur	1667 (34.5)	738 (44.3)	929 (55.7)	-	551 (37.6)	345 (62.6)	206 (37.4)	-
Shoulder/humerus/scapula/rib/sternum/skull	1216 (25.2)	449 (36.9)	767 (63.1)	-	423 (28.9)	240 (56.7)	183 (43.3)	-
Spine	1949 (40.3)	1028 (52.7)	921 (47.3)	-	491 (33.5)	383 (78.0)	108 (22.0)	-
ECOG ≥ 2				<.0001				.7617
<2	2937 (66.9)	1418 (48.3)	1519 (51.7)	-	977 (72)	641 (65.6)	336 (34.4)	-
≥2	1451 (33.1)	578 (39.8)	873 (60.2)	-	380 (28)	246 (64.7)	134 (35.3)	-
Fatigue at baseline, grade				.0002				.00742
0-1	3379 (73.5)	1613 (47.7)	1766 (52.3)	-	1049 (74.1)	707 (67.4)	342 (32.6)	-
≥2	1217 (26.5)	494 (40.6)	723 (59.4)	-	367 (25.9)	219 (59.7)	148 (40.3)	-
Systemic therapy <4 wk prior to RT				.164				.16501
No	2070 (43.0)	972 (47.0)	1098 (53.0)	-	558 (38.2)	357 (64.0)	201 (36.0)	-
Yes	2746 (57.0)	1234 (44.9)	1512 (55.1)	-	902 (61.8)	609 (67.5)	293 (32.5)	-
CNS or visceral disease				.1219				.01355
No	1934 (41.8)	905 (46.8)	1029 (53.2)	-	694 (48.1)	481 (69.3)	213 (30.7)	-
Yes	2690 (58.2)	1197 (44.5)	1493 (55.5)	-	749 (51.9)	473 (63.2)	276 (36.8)	-
No. of Regions treated				.9195				.1015
1	2676 (56.6)	1226 (45.8)	1450 (54.2)	-	847 (57.8)	545 (64.3)	302 (35.7)	-
≥2	2054 (43.4)	938 (45.7)	1116 (54.3)	-	618 (42.2)	423 (68.4)	195 (31.6)	-
Retreatment				.0002				-
No	4345 (89.9)	2031 (46.7)	2314 (53.3)	-	1465 (100)	968 (66.1)	497 (33.9)	-
Yes	487 (10.1)	184 (37.8)	303 (62.2)	-	-	-	-	-
**Facility characteristics**								
Facility type				.0386				.05306
Nonacademic	3818 (79.0)	1721 (45.1)	2097 (54.9)	-	1215 (82.9)	816 (67.2)	399 (32.8)	-
Academic	1014 (21.0)	494 (48.7)	520 (51.3)	-	250 (17.1)	152 (60.8)	98 (39.2)	-
Physician’s years of practice				<.0001	304 (20.8)	171 (56.3)	133 (43.8)	.00025
0-10	1399 (29.0)	569 (40.7)	830 (59.3)	-	466 (31.8)	307 (65.9)	159 (34.1)	-
11-20	1371 (28.4)	592 (43.2)	779 (56.8)	-	316 (21.6)	224 (70.9)	92 (29.1)	-
21-30	875 (18.1)	448 (51.2)	427 (48.8)	-	379 (25.9)	266 (70.2)	113 (29.8)	-
>30	1187 (24.6)	606 (51.1)	581 (48.9)	-	304 (20.8)	171 (56.3)	133 (43.8)	-

Missing data are excluded. P values were generated via the χ^2^ test.

*Abbreviations:* CNS = central nervous system; ECOG = Eastern Cooperative Oncology Group; NSCLC = non-small cell lung cancer; PFS = progression-free survival; RT = radiation therapy; SCLC = small cell lung cancer.

**Figure 1:**
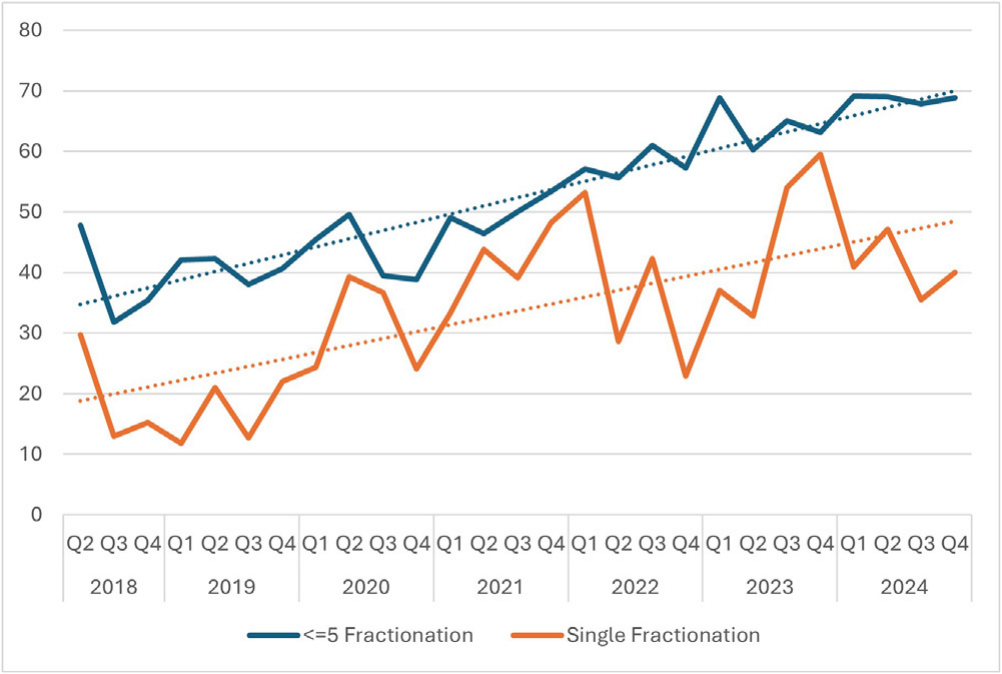
Trends in the percentage of radiation therapy (RT) plans delivering ≤5 fractions and single fractions during the observation period. *Abbreviations:* Q = quarter.

Figure 1 illustrates the use of SFRT and ≤5-fraction RT over time. Both overall trends were positive during the observation period, with quarterly and annual variation. The use of SFRT for uncomplicated metastases increased from 17.8% prior to 2020 to 38.8% after the implementation of the quality measure, while the use of ≤5-fraction RT increased from 44.2% prior to 2022 to 63.9% after the implementation of the quality measure.

The most common fractionations among uncomplicated metastases were 8 Gy in 1 fraction (455 patients, 31.1%), 30 Gy in 10 fractions (389 patients, 26.6%), and 20 Gy in 5 fractions (386 patients, 26.3%). Among all patients included in the analysis for ≤5-fraction RT, the most common fractionations were 30 Gy in 10 fractions (1763 patients, 36.5%), 20 Gy in 5 fractions (1381 patients, 28.6%), and 8 Gy in 1 fraction (980 patients, 20.3%). All other fractionations accounted for less than 5% of each cohort.

Multivariable analyses of factors associated with SFRT among uncomplicated metastases and ≤5-fraction RT among all bone metastases are presented in Table 2, Table 3, respectively. After adjusting for covariates and clustering by center, both models demonstrated that the year of treatment (either as a continuous or categorical variable) was significantly associated with the use of SFRT and ≤5-fraction RT. In both models, a longer distance to the treating facility, higher baseline fatigue, treatment site other than the spine, and fewer physician years of practice predicted shorter treatment courses. Among uncomplicated metastases, patients requiring treatment at more than 1 site had lower odds of receiving SFRT. After adjusting for clustering, neither primary histology nor planning type was associated with a shorter treatment course for uncomplicated metastases. However, among all metastases, there were lower odds of ≤5-fraction RT for breast histology and higher odds of ≤5-fraction RT with forward planning. Uncomplicated metastases, retreatment, and palliative intent independently predicted the use of ≤5-fraction RT. Patients aged >80 years and those with an Eastern Cooperative Oncology Group performance status ≥2 were more likely to receive ≤5-fraction RT.

**Table 2:** Multivariable logistic regression and random intercept analysis to adjust for clustering of factors associated with receipt of single-fraction radiation therapy among patients with uncomplicated bone metastases

	Multivariable analysis				Random intercept analysis			
Variable	OR	95% CI		*P* value	OR	95% CI		*P* value
Year 2018-2019 2020-2024	(Ref) 3.327	2.392	4.626	<.0001	(Ref) 12.142	5.081	29.015	<.0001
Primary malignancy Lung Breast Prostate Renal cell carcinoma	(Ref) 0.709 1.146 1.095	0.528 0.851 0.623	0.952 1.544 1.926	.0055 .1473 .563	-	-	-	-
Distance to treatment center (continuous, unit 10 miles)	1.094	1.029	1.162	.0041	1.261	1.09	1.458	.0018
Planning type Inverse planning Forward planning	(Ref) 1.736	1.183	2.549	.0048	-	-	-	-
Anatomic site treated Spine Hip/pelvis/femur Shoulder/humerus/rib/skull/other	(Ref) 2.286 3.206	1.695 2.338	3.083 4.397	.0495 <.0001	(Ref) 5.367 9.526	3.009 4.847	9.57 18.72	<.0001 <.0001
Fatigue at baseline, grade 0-1 ≥2	(Ref) 1.53	1.169	2.003	.0019	(Ref) 2.325	1.246	4.336	.0081
No. of regions treated 1 ≥2	(Ref) 0.714	0.558	0.912	.0071	(Ref) 0.397	0.223	0.704	.0016
Physician’s years of practice 0-10 11-20 21-30 >30	(Ref) 0.61 0.586 0.556	0.44 0.406 0.392	0.847 0.846 0.788	.3577 .2515 .0846	(Ref) 0.442 0.45 0.337	0.198 0.184 0.149	0.988 1.1 0.764	.0466 .0799 .0093

Lung primary malignancy includes both small cell and non-small cell lung cancer. Melanoma was excluded from the analysis due to the small number of patients representing this primary site.

*Abbreviations:* OR = odds ratio; Ref = reference.

**Table 3:** Multivariable logistic regression and random intercept analysis to adjust for clustering of factors associated with receipt of ≤5-fraction radiation therapy

	Multivariable analysis				Random intercept analysis			
Variable	OR	95% CI		*P* value	OR	95% CI		*P* value
Year (continuous)	1.211	1.118	1.312	<.0001	1.508	1.403	1.621	<.0001
Year 2018-2021 2022-2024	(Ref) 1.395	1.054	1.846	.02	-	-	-	-
Age ≤59 60-69 70-79 80+	(Ref) 1.245 1.224 1.729	1.028 0.999 1.346	1.508 1.499 2.22	.6926 .5112 .0001	(Ref) 1.221 1.2 1.898	0.901 0.878 1.294	1.653 1.639 2.783	.1971 .2522 .0011
Primary malignancy Lung Breast Melanoma Prostate Renal cell carcinoma	(Ref) 0.753 1.301 0.831 0.803	0.632 0.749 0.685 0.605	0.899 2.257 1.008 1.067	.0163 .1185 .2668 .2644	(Ref) 0.67 1.241 0.765 0.679	0.503 0.51 0.573 0.453	0.892 3.023 1.021 1.019	.0062 .634 .0689 .0615
Distance to treatment center (continuous, unit of 10 miles)	1.045	1.013	1.078	.0056	1.072	1.021	1.125	.0049
Planning type Inverse planning Forward planning	(Ref) 1.678	1.299	2.169	<.0001	(Ref) 2.143	1.499	3.064	<.0001
Uncomplicated metastasis No Yes	(Ref) 1.906	1.585	2.292	<.0001	(Ref) 2.478	1.882	3.263	<.0001
Treatment intent Palliation for existing symptoms Curative intent or improvements in PFS Durable local control Treatment of existing pathologic fracture Treatment of spinal cord compression	(Ref) 0.913 0.59 0.444 0.581	0.496 0.481 0.333 0.429	1.678 0.723 0.593 0.787	.2228 .1775 .0013 .2711	(Ref) 0.991 0.499 0.317 0.344	0.4 0.37 0.209 0.204	2.454 0.673 0.48 0.581	.9842 <.0001 <.0001 <.0001
Anatomic site treated Spine Hip/pelvis/femur Shoulder/humerus/rib/skull/other	(Ref) 1.408 1.997	1.192 1.656	1.663 2.408	.9592 <.0001	(Ref) 1.651 2.524	1.343 1.969	2.03 3.234	<.0001 <.0001
Fatigue at baseline, grade 0-1 ≥2	(Ref) 1.573	1.332	1.858	<.0001	(Ref) 1.689	1.307	2.182	<.0001
ECOG 0-1 ≥2	(Ref) 1.275	1.089	1.494	.0026	(Ref) 1.484	1.182	1.862	.0007
Retreatment No Yes	(Ref) 1.879	1.464	2.413	<.0001	(Ref) 2.058	1.474	2.874	<.0001
Physician’s years of practice 0-10 11-20 21-30 >30	(Ref) 0.842 0.653 0.611	0.695 0.525 0.501	1.02 0.812 0.743	.0982 .031 .0005	(Ref) 0.834 0.607 0.538	0.612 0.425 0.394	1.138 0.866 0.736	.2522 .0059 .0001

Lung primary malignancy includes both small cell and non-small cell lung cancer.

*Abbreviations:* ECOG = Eastern Cooperative Oncology Group; OR = odds ratio; PFS = progression-free survival; Ref = reference.

## Discussion

These results highlight the complexity of decision-making in the treatment of bone metastases. We continue to observe varied practice within the statewide quality consortium; however, we have noted a trend over time toward the use of fewer fractions for RT of bone metastases, consistent with the consortium’s quality goals. We previously reported that SFRT use increased from 15% to 28% among patients with uncomplicated bone metastases within the first year of metric introduction.^16^

While not independently significant after adjusting for clustering, the significance of year as both a continuous and a categorical (before vs after quality measure introduction) variable in multivariable analysis for the use of 5 or fewer fractions suggests a pre-existing time trend driving increased adoption of shorter treatments, and the measure was associated with an additional, immediate increase in use beyond what would be expected from the trend alone. Our observation period included the COVID-19 pandemic, and the SFRT quality initiative launched just months before the pandemic impacted daily practice. The COVID-19 pandemic was a driver of hypofractionation use across multiple disease sites, including bone metastases, to reduce possible patient exposure to the novel coronavirus.^22^, ^23^, ^24^ Some evidence suggests a trend toward increased use of fewer fractions for bone metastasis predating the pandemic, with 1 institution in our state reporting an estimated inflection point in 2015.^25^ It is difficult to account for the impact of COVID-19 on fractionation in our consortium and to fully interpret the significance of treatment year in multivariable analyses, although the pandemic is reasonably considered a driver of the use of fewer fractions in the consortium, alongside implemented quality metrics.

Initiatives that support the use of fewer treatment fractions are designed to promote patient convenience, reduce financial toxicity (which has been shown to affect about half of patients receiving palliative RT), decrease the environmental impact of treatment, and support the judicious use of health care resources.^11^, ^12^, ^13^, ^14^ Given this context, it is unsurprising that patients who traveled farther were more likely to receive 5 or fewer fractions or SFRT in our analyses. Similarly, patients with higher baseline fatigue were more likely to receive fewer fractions. Notably, treatment facility type (academic vs nonacademic) was not independently associated with fractionation. Absolute differences in fractionation selection by facility type were small (<10%) in both analyses, highlighting the effective introduction of quality initiatives and incentive structures across varied practice settings.

We observed that patients undergoing reirradiation (reRT) were more likely to receive 5 or fewer fractions. With continuous improvements in cancer management and patients living longer with cancer, consideration of reRT is expected to increasingly drive decision-making and is relevant not only in selecting fractionation to optimize OAR sparing versus target dose, but also in the upfront setting, with consideration of the durability of response and the ability to retreat the same location following initial fractionation.^1^^,^^26^, ^27^, ^28^

In all patients, forward planning was associated with higher odds of receiving 5 or fewer fractions, independent of the presence of uncomplicated metastases. This is difficult to further characterize or explain with the available data. Forward planning may be used to deliver SFRT to both complicated and uncomplicated metastases, the latter of which was a well-represented fractionation in this cohort. Similarly, the use of forward planning and fewer fractions together can aid the delivery of expedient RT for palliation, which was a well-represented indication for RT in the cohort assessed for ≤5-fraction treatment.

In our assessment of the use of 5 or fewer fractions among all patients, SBRT plans were excluded, as they were expected to be delivered in 5 or fewer fractions. Given the incentive structure to reward quality consortium participation and meeting quality metrics, some practices have been granted exemptions from BCBSM prior authorization. We do not here characterize the use of SBRT in this structure, although this would be expected to confound any analysis and limit generalizability to the broader United States. Given the exclusion of SBRT plans from our ≤5-fraction analysis and observations of increased SBRT use over time in our consortium, overall rates of plans delivering 5 or fewer fractions are higher than reported in this manuscript, especially in more recent years.

Stereotactic approaches may be used more frequently in patients with oligometastatic disease to prevent skeletal events or to decrease the planning treatment volume margin in the setting of reRT or adjacent critical OARs. With the increasing role of stereotactic approaches, the inclusion of stereotactic treatments in future analyses will be important, including examining treatment intent and characterizing the use of appropriate OAR delineation and constraints in stereotactic plans.

Notably, histology did predict the use of ≤5 fractions in our analysis; higher biologically effective doses afforded by treatments exceeding 5 fractions or stereotactic approaches may be differentially preferred for radioresistant histology.^1^ Patient sex was not included in our multivariable models, with the primary site being the more likely explanation for differences in treatment by sex, given prostate cancer is sex-specific, and breast cancer predominantly occurs in females. Additionally, the predisposition to micrometastatic and/or polymetastatic states by histology (eg, breast cancer) or prognosis, and the availability of next-line systemic therapy in these states by histology, may influence provider decision-making. While we assessed the number of regions treated in each episode, this does not necessarily reflect the total systemic disease burden.

A postpandemic quality initiative involving knowledge dissemination, personalized feedback, and eConsult availability, but without payer involvement, demonstrated improvement over time; however, the postimplementation timeframe was not independently associated with adoption of shorter fractionation.^29^ Conversely, knowledge transfer campaigns have effectively increased the use of SFRT for bone metastasis within the Canadian health system.^30^ The MROQC has established a symbiotic partnership between payers and providers to deliver incentivized, high-quality, high-value care to cancer patients in the state of Michigan and may serve as a model for delivery in other states. Strategic inclusion of fractionation and other dosimetric considerations in quality metrics and knowledge outputs from the consortia spans all cancer types included in the consortium.^19^^,^^31^, ^32^, ^33^, ^34^ Metrics within the consortium are measured for all enrolled patients, not just those insured by BCBSM. Similar initiatives span multiple disciplines in our state, suggesting that quality consortium models like MROQC’s have applicability beyond radiation oncology.^35^, ^36^, ^37^

Limitations of these data include patients enrolled at participating facilities who were limited to a single state in the continental United States. However, with prospectively collected data from a large cohort of patients in a state that is geographically, socioeconomically, and racially diverse, we anticipate that these data will be useful beyond the geographic borders of our quality consortium.

## Conclusions

We observed that radiation fractionation is complex and multifactorial, reflecting the intricacies involved in decision-making for the treatment of bone metastases with RT. We observed an increase in the use of fewer fractions over time, consistent with the successful implementation of quality measures and the sustained influence on practice within the consortium’s incentive structure.

## Disclosures

Luke M. Higgins reports travel funding from the American Society for Radiation Oncology. Huiying (Maggie) Yin, Kent Griffith, Jumoke Johnson-Olokesusi, Lana Critchfield, and James A. Hayman receive salary support from Blue Cross Blue Shield of Michigan through the Michigan Radiation Oncology Quality Consortium.
